# ATP translocation and chloroplast biology

**DOI:** 10.1093/nsr/nwz089

**Published:** 2019-07-12

**Authors:** Chia P Voon, Boon L Lim

**Affiliations:** School of Biological Sciences, University of Hong Kong, China

By studying changes in ATP levels in the plastids and cytosol of live plants using a MgATP^2^-specific Förster resonance energy transfer (FRET)-based sensor, the entry of cytosolic ATP to the mature chloroplasts of *Arabidopsis thaliana* was found to be negligible [1]. That ATP can be translocated into and out of mature plant chloroplast was first reported in 1969 [2]. Since then, it has been suggested that cytosolic ATP, mainly generated from the respiratory chain of the mitochondria, can enter chloroplasts to fulfil its energy demand at night [3–5].

In Heldt *et al.* [2], spinach chloroplasts were first incubated with ^14^C-ATP for 1 hour at 0°C to allow ATP uptake. After the chloroplasts were washed twice, unlabeled ATP/ADP/AMP/NTPs were added to the suspension. The amount of radioactive ^14^C-label released to the supernatant was then measured and this was regarded as ^14^C-ATP exchanged from the chloroplasts. Unlabeled ATP was the most effective agent to exchange ^14^C-ATP and the exchanged activity was decreased to 12% when unlabeled ADP was used [2]. However, the plastidic nucleotide transporter (NTT) is an ATP/ADP exchanger [3,6]. Therefore, if NTTs functioned in mature spinach chloroplasts, then unlabeled exogenous ADP should have been more effective than ATP to exchange with ^14^C-ATP inside the chloroplasts. Since this was not the case, it is more likely that ^14^C-ATP was absorbed on the surface of the organelles rather than imported. Absorbed ^14^C-ATP was readily displaced by unlabeled ATP, and by ADP to a lesser extent [2]. Hence, these experimental data suggest that ATP did not translocate across the envelop of mature spinach chloroplasts.

## MATURE CHLOROPLASTS DO NOT IMPORT ATP EFFICIENTLY

**Figure 1.**
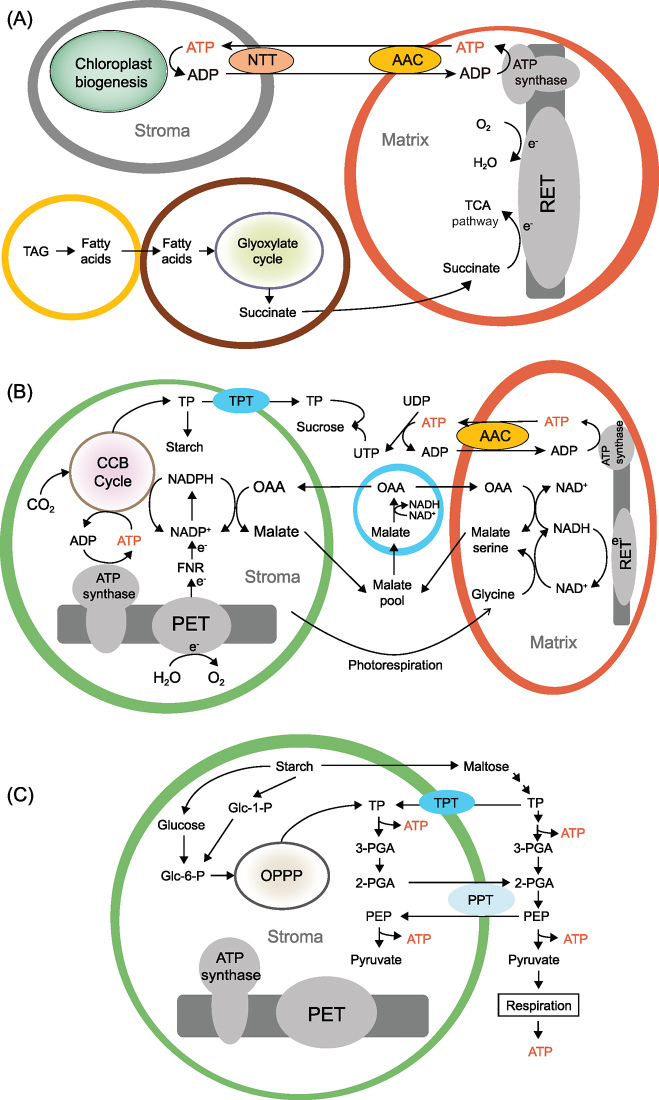
Three different scenarios of chloroplast bioenergetics in *Arabidopsis thaliana*. (A) During the transition of proplastid (grey) to chloroplast, cytosolic ATP is imported into the chloroplast through the NTT. ATP is mostly generated in mitochondria (red) using succinate derived from TAGs. TAG is stored in lipid bodies (yellow) and catabolized into fatty acids before its transportation into glyoxysome (brown), where fatty acids are converted to succinate through the glyoxylate cycle. (B) In mature chloroplast, NTT is down-regulated to restrict ATP import from cytosol. To balance the ATP:NADPH demand and supply during photosynthesis, surplus-reducing equivalents from the LEF are exported to extrachloroplast compartments. Mitochondria can dissipate excess reducing equivalents from the chloroplasts and supply ATP to the cytosol for sucrose synthesis. The sucrose produced during photosynthesis can be delivered to sink tissues. The blue cycle is a peroxisome. (C) At night, starch is degraded into maltose, glucose and glucose-1-phosphate (Glc-1-P). While maltose is exported to the cytosol, the latter two can be converted to TP through the OPPP. It should be noted that chloroplasts lack enolase (ENO1), the enzyme that converts 2PGA to PEP [14]. Hence, PEP has to be imported from the cytosol via the PPT. Pyruvate produced by the plastidic glycolytic pathway is the substrate for multiple pathways, including the biosynthesis of isoprenoids, branched-chain amino acids and fatty acids [3]. AAC, ADP/ATP carrier protein; FNR, ferredoxin-NADP^+^ oxidoreductase; OAA, oxaloacetate; OPPP, oxidative pentose phosphate pathway; PEP, phosphoenolpyruvate; PET, photosynthetic electron-transport chain; PGA, phosphoglycerate; PPT, phosphoenolpyruvate/phosphate translocator; RET, respiratory electron-transport chain; TAGs, triacylglycerols; TCA, tricarboxylic acid; TP, triose phosphate; TPT, triose phosphate/phosphate translocator.

An ATP sensor introduced into the stroma and cytosol of *Arabidopsis* revealed that ATP concentrations in these compartments were similar in young seedlings (3–4 days old), whereas, in 10-day-old cotyledons, the stromal ATP concentration decreased to a level significantly lower than in the cytosol [1]. Moreover, exogenous ATP was able to enter young chloroplasts isolated from 4- and 5-day-old seedlings but was unable to enter chloroplasts isolated from 10-day-old cotyledons or 20-day-old leaves. In line with this result, promoter-GUS studies showed that *NTT*s are highly expressed in young seedlings but not in older seedlings [7]. We proposed that ATP import is required in young seedlings to meet the energy demands during chloroplast biogenesis (Fig. 1A).

## CHLOROPLASTS ARE ALWAYS STARVED OF ATP

Illumination transiently increased the stromal ATP concentration in mature mesophyll but it then dropped to a baseline level within 30 sec after the light was turned off [1]. Transfection of the *NTT*s in protoplasts also drained cytosolic ATP. Hence, both observations indicate that mature chloroplasts consume ATP rapidly. Cellular ATP is mainly generated from mitochondria, which is bioenergetically active immediately upon imbibition [8]. In *Arabidopsis thaliana*, the substrate for mitochondrial respiration during germination is mainly derived from the stored oil bodies [9] (Fig. 1A). Once chloroplasts become photosynthesis-competent, ATP import is terminated by the down-regulation of *NTT*s (Fig. 1B and C). This design could allow mesophyll to reduce the ATP consumption in the chloroplasts at night.

## EXPORT OF REDUCING EQUIVALENTS IS IMPORTANT FOR PHOTOSYNTHESIS

Chloroplast photosystems generate ATP and NADPH during photosynthesis. To fix one CO_2_ molecule, three ATP molecules and two NADPH molecules (ATP/NADPH = 1.5) are consumed by the Calvin-Benson-Bassham (CBB) cycle (Fig. 1B). However, the linear electron flow (LEF) only generates ATP and NADPH at a ratio of 1.28. While other alternative pathways such as cyclic electron transport around PSI can generate additional ATP [10], recent results [1] imply that, instead of ATP import, the export of reducing equivalents from chloroplasts is important for balancing the ATP/NADPH demand of the CBB cycle and the ATP/NADPH supply from the LEF (Fig. 1B). Because the demand of NADPH/ATP for carbon dioxide fixation is approximately at a ratio of 0.67, and 0.78 molecules of NADPH are generated from each ATP molecule in the LEF [11], one could expect that surplus-reducing equivalents are generated from the LEF under prolonged illumination [10]. To recycle NADP^+^ for efficient photosynthesis, the surplus-reducing equivalents must be dissipated or exported (Fig. 1B). By studying transgenic plants with a pH-sensor protein targeted to the mitochondrial matrix, photosynthesis was found to be responsible for the rapid light-induced changes in the matrix pH, suggesting that mitochondria play an important role in consuming the reducing equivalents generated from photosynthesis for ATP synthesis [1]. Moreover, when the mitochondrial electron-transport chain was inhibited, the rate of ATP production in chloroplasts was reduced [1].

## WHAT IS THE SOURCE OF ATP IN CHLOROPLASTS AT NIGHT?

Heterotrophic plastids rely on the import of cytosolic ATP and carbon to drive metabolic activities [6]. These plastids can also generate ATP through the plastidial glycolysis of glucose 6-phosphate (Glc-6-P) [12], which is derived from sucrose delivered from source tissues [3]. For chloroplasts, the nocturnal import of ATP was suggested as an alternative to photosynthesis to supply ATP at night [3–5]. A double *ntt* null mutant exhibited retarded growth and diminished starch accumulation under short photoperiod/low irradiance (125 μE) but not under long-photoperiod/low-irradiance conditions. These phenotypes led to the conclusion that nocturnal import of ATP to chloroplasts was important [4]. However, recent results indicate that ATP import into mature chloroplasts is negligible [1]. Therefore, it is more likely that the *ntt* mutant phenotypes stem from the lack of ATP import during chloroplast biogenesis (Fig. 1A) [7], which might diminish starch accumulation in chloroplasts during the short photoperiod and/or a defect in ATP import into the heterotrophic plastids (e.g. root plastids). Carbon in the form of plastidial starch is an important reservoir of energy in chloroplasts. Starch is mobilized via glycolytic steps in the stroma that can regenerate ATP at night [5] (Fig. 1C). Under short-photoperiod and low-irradiance conditions, the lower starch level in *ntt* chloroplasts ultimately leads to higher protoporphyrin IX (Proto IX) accumulation, reactive oxygen species production and retarded growth [4]. The key step of chlorophyll synthesis—the insertion of Mg^2+^ into Proto IX by Mg chelatase—is an ATP-dependent process [13]. The accumulation of Proto IX in *ntt* mutant could be due to a suboptimal stromal ATP level during the night. These *ntt* phenotypes are minimized under long-photoperiod/low-irradiance or short-photoperiod/high-irradiance (400 μE) conditions when the mutants accumulated sufficient starch to support ATP expenditure at night [4]. When the ATP level is low, more carbon consumption via glycolysis would be expected to catch up with the energy shortfall. This idea is also supported by the observation that transcripts for three plastidial pyruvate kinase isoforms, one of the key ATP-generating enzymes in glycolysis, are substantially higher in *ntt* null mutants [4]. Collectively, plastidial glycolysis of the starch reservoir is the source of ATP in chloroplasts at night (Fig. 1C).

## CONCLUDING REMARKS

In higher plants, mesophyll chloroplasts play an essential role in supplying sugars to developing tissues when light is available. Before they are photosynthetically active, the ATP demand of chloroplast biogenesis must be met by the import of ATP from the cytosol via NTTs (Fig. 1A). However, chloroplasts could also consume a large amount of energy at night. We propose that, during the evolution of unicellular to multicellular photosynthetic organisms, the flux of ATP between the cytosol and stroma of mature chloroplasts was restricted by the down-regulation of *NTT* expression to control the energy expenditure and chloroplast activities at night (Fig. 1B and C). Thus, mature chloroplasts can be more energy-efficient.
